# Zika virus-induced acute myelitis and motor deficits in adult interferon αβ/γ receptor knockout mice

**DOI:** 10.1007/s13365-017-0595-z

**Published:** 2018-02-23

**Authors:** Katherine Zukor, Hong Wang, Venkatraman Siddharthan, Justin G. Julander, John D. Morrey

**Affiliations:** Institute for Antiviral Research, Department of Animal, Dairy, and Veterinary Sciences, Utah State University, 5600 Old Main Hill, Logan, UT 84322-5600 USA

**Keywords:** Zika virus, Brain, Spinal cord, Motor, Guillian-Barrë syndrome, Mouse

## Abstract

Zika virus (ZIKV) has received widespread attention because of its effect on the developing fetus. It is becoming apparent, however, that severe neurological sequelae, such as Guillian-Barrë syndrome (GBS), myelitis, encephalitis, and seizures can occur after infection of adults. This study demonstrates that a contemporary strain of ZIKV can widely infect astrocytes and neurons in the brain and spinal cord of adult, interferon α/β receptor knockout mice (AG129 strain) and cause progressive hindlimb paralysis, as well as severe seizure-like activity during the acute phase of disease. The severity of hindlimb motor deficits correlated with increased numbers of ZIKV-infected lumbosacral spinal motor neurons and decreased numbers of spinal motor neurons. Electrophysiological compound muscle action potential (CMAP) amplitudes in response to stimulation of the lumbosacral spinal cord were reduced when obvious motor deficits were present. ZIKV immunoreactivity was high, intense, and obvious in tissue sections of the brain and spinal cord. Infection in the brain and spinal cord was also associated with astrogliosis as well as T cell and neutrophil infiltration. CMAP and histological analysis indicated that peripheral nerve and muscle functions were intact. Consequently, motor deficits in these circumstances appear to be primarily due to myelitis and possibly encephalitis as opposed to a peripheral neuropathy or a GBS-like syndrome. Thus, acute ZIKV infection of adult AG129 mice may be a useful model for ZIKV-induced myelitis, encephalitis, and seizure activity.

**Electronic supplementary material:**

The online version of this article (10.1007/s13365-017-0595-z) contains supplementary material, which is available to authorized users.

## Introduction

Zika virus (ZIKV) is an emerging flavivirus that has received widespread attention because of its effect on the developing fetus. In utero infections cause congenital defects, most notably microcephaly along with other deformities (Schuler-Faccini et al. 2016). While ZIKV infection of adults generally produces only a mild disease, it is becoming apparent that, as with other flavivirus infections, severe neurological sequelae can occur. Recent outbreaks have been associated with higher incidences of peripheral neuropathies such as Guillian-Barrë syndrome (GBS) (Brasil et al. 2016; Cao-Lormeau et al. 2016; Cardoso et al. 2015; Samarasekera and Triunfol 2016) and other neurological diseases such as myelitis (Anaya et al. 2017; Dirlikov et al. 2016; Mecharles et al. 2016), encephalitis (Carteaux et al. 2016; Nicastri et al. 2016; Soares et al. 2016), seizures (Asadi-Pooya 2016), and various ophthalmological conditions (Pastula et al. 2016; Smith et al. 2016). This is not surprising considering that related flaviviruses such as West Nile virus (WNV) and Japanese encephalitis virus (Solomon et al. 1998) can cause myelitis and motor deficits (Sejvar et al. 2003). Given the emerging nature of ZIKV, however, it is likely that we do not fully understand the acute and long-term consequences of ZIKV infection on the nervous system. Therefore, investigating the pathobiology of ZIKV in animal models will help to understand the potential for ZIKV to cause neurological disease in human subjects. We focus herein on adult models because ZIKV-related motor deficits have been primarily associated with adult infection, as opposed to in utero infection (Anaya et al. 2017; Dirlikov et al. 2016; Mecharles et al. 2016).

Prior to the 2015 ZIKV outbreak, a few animal models of ZIKV infection existed, but infection of rodents was largely non-productive unless a lab strain of the virus that had undergone several serial passages in mice was used (Dick 1952). After the recent outbreaks, efforts to develop animal models with more clinically relevant isolates of the virus have been renewed. A rapid series of publications found that adult mice that lack type 1 (αβ; A129 and IFNAR^−/−^ strains) or types 1 and 2 (αβ/γ; AG129 strain) interferon receptors are susceptible to lethal infection (Aliota et al. 2016; Lazear et al. 2016; Manangeeswaran et al. 2016; Rossi et al. 2016; Zmurko et al. 2016). These models may have some relevance to human ZIKV infections in that, like many viruses, ZIKV gains advantages in human hosts by inhibiting interferon responses (Best 2017; Bowen et al. 2017; Schulz and Mossman 2016). Because viruses may not be able to inhibit mouse-specific interferon pathways (Aguirre et al. 2012), blocking them by other means, as in AG129 mice, may more closely mimic what happens in humans. Although AG129 mice are deficient in innate immune responses, they do elicit acquired immune responses, such as vaccine-elicited protective immunity (Sumathy et al. 2017; Weger-Lucarelli et al. 2017). While complete knockout of the interferon responses produces a more severe disease in mice, it can provide insights into what happens when ZIKV gains access to the adult central nervous system (CNS). Besides a lethal infection, these mice manifest neurological symptoms such as “toe walking,” tremors, loss of balance, paralysis, and hunched posture (Aliota et al. 2016; Lazear et al. 2016; Manangeeswaran et al. 2016; Rossi et al. 2016).

To better understand the consequences of ZIKV infection of the adult nervous system and the pathobiology of the resulting neurological disease, we infected AG129 mice with a contemporary, low-passage ZIKV isolate and evaluated the neurological motor deficits occurring during the acute phase of the disease. We characterized the onset, course, and severity of behavioral hindlimb deficits and used electrophysiology and immunohistochemistry (IHC) to determine if such deficits are due primarily to myelitis, peripheral neuropathy, myositis, or encephalitis. This included histological analysis of motor cortex, spinal cord, peripheral nerve, and muscle.

## Materials and methods

### Animals

Male and female AG129 mice (van den Broek et al. 1995) were bred in-house in sterilized cages and maintained in a 12/12 light cycle. Mice were randomly assigned to treatment groups based on weight, gender, and baseline measurements.

### Virus

A Puerto Rican isolate of ZIKV (PRVABC59, Human/2015/Puerto Rico, GenBank KU501215) was obtained from BEI Resources (Cat No. NR-50240, Lot No. 64112564). The certificate of analysis confirmed that the sequence of this stock (GenBank KX087101) is 99% identical to PRVABC59 (GenBank KU501215). The BEI stock was passaged two times in Vero 76 cells to make a stock with a titer of 2 × 10^7^ pfu/mL for use in all experiments. Infected cells were frozen once, thawed, centrifuged to remove cell debris, and aliquoted in frozen stocks. Dilutions were made in minimal essential medium supplemented with 50 μg/mL gentamicin to deliver 2000 pfu subcutaneously in the inguinal area on the right side in a volume of 0.1 mL. Uninfected cells were prepared and diluted similarly for sham infections.

### Experiment no. 1

**Fig. 1:**
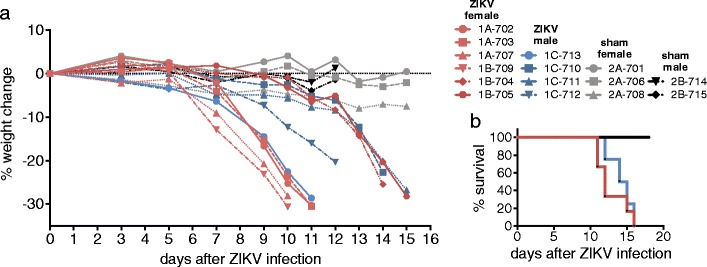
Weight loss and survival curves for AG129 adult mice infected with ZIKV in experiment no. 1. **a** Body weight expressed as a percentage of starting weight after ZIKV or sham infection. **b** Percentage of each group surviving at each day after ZIKV or sham infection. All sham-infected animals (male and female) are represented in the sham group

**Fig. 2:**
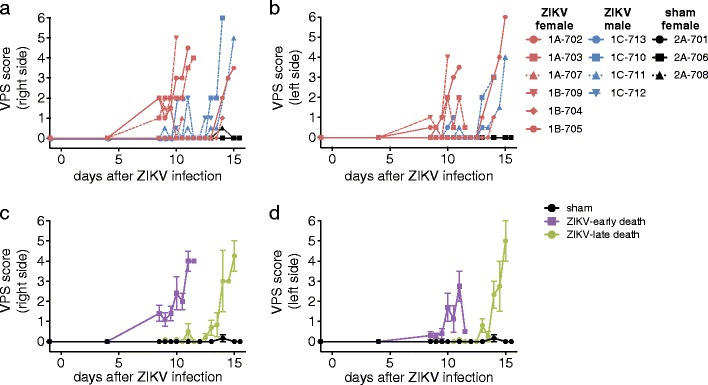
AG129 mice infected with ZIKV experience moderate paresis to severe paralysis before death. **a**, **b** VPS scores from experiment no. 1 of individual mice on the right (**a**) and left sides (**b**). **c**, **d** Mean VPS scores of ZIKV-infected mice that died early (*n* = 5) or late (*n* = 5) compared to sham-infected mice (*n* = 3) on the right (**c**) and left sides (**d**). Error bars represent SEM

**Fig. 3:**
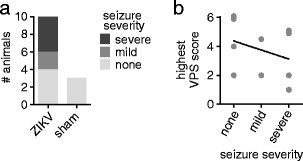
Seizure activity occurs independently of hindlimb motor deficit level. **a** Number of animals in sham-infected or ZKIV-infected groups from experiment no. 1 having mild or severe seizure activity. **b** Correlation between highest VPS score and seizure severity for ZIKV-infected animals. Each dot represents one animal. The line is the best fit line if 0, 1, and 2 are used for seizure categories of none, mild, and severe, respectively. Slope is not significantly different from zero

The purpose of this experiment was to assess the time course and severity of motor deficits and collect tissues for histological analysis. Seventy-two to 75-day-old (10.5 weeks) male and female AG129 mice were infected with ZIKV (*n* = 6 females and 4 males) or sham (*n* = 3 females and 2 males) inoculum and monitored for weight loss and survival (Fig. 1). Behavioral motor assessments were performed before infection (baseline); once after infection, but before symptom onset (4 days post-infection (DPI)); and twice a day after symptom onset (9–15 DPI) (Fig. 2). Seizure-like activity observed during behavioral assessments was also noted (Fig. 3). Videos of motor deficits and seizure-like activity were obtained to provide examples of symptoms seen. Observations were made by researchers who were blind to the infection status of each group. Moribund mice were perfused for histological analysis of the brain, lumbosacral spinal cord, sciatic nerve, and gastrocnemius muscle.

### Experiment no. 2

The purpose of this experiment was to collect electrophysiological data on mice with motor deficits. Male and female AG129 mice aged 120–123 days old (17 weeks) or 62 days old (9 weeks) were divided into groups. The ZIKV-infected group contained three males (17 weeks old), four males (9 weeks old), and four females (9 weeks old). The sham-infected group contained two males (17 weeks old), two males (9 weeks old), and two females (9 weeks old). Mice were monitored for weight loss and survival. Behavioral assessments were performed before infection (baseline) and after symptom onset so that mice with moderate to severe deficits (scores 3–6, see below) could undergo electrophysiological assessment. Compound muscle action potential (CMAP) of the gastrocnemius muscle was recorded before infection (baseline) and after moderate to severe deficits had developed. After infection, one sham-infected mouse was recorded for every one to two ZIKV-infected mouse. Measurements were obtained by a researcher who was blind to the infection status and deficit score of each mouse. CMAP responses were recorded in response to stimulation at the sciatic notch and then the lumbosacral spinal cord. Because stimulation of the lumbar spinal cord was an invasive procedure, we did not obtain baseline measurements for CMAP in response to spinal cord stimulation.

### Viral paresis scale

Mice were analyzed for signs of tail and hindlimb paresis/paralysis using a sensitive, open-field assay modified from the Basso Mouse Scale used to assess paralysis in spinal cord-injured mice (Basso et al. 2006) and a test used to track paralysis in amyotrophic lateral sclerosis mouse models (Hatzipetros et al. 2015). Each mouse was placed on a tabletop and allowed to roam freely for 4 min. Hindlimb function was scored on a seven-point scale detailed in Table 1 by researchers who were blind to the infection status of each group. Scoring was based on four main categories: tail position during walking, miss-step severity, weight bearing, and joint movement. Miss-step severity was scored only on assessable walking passes, which was defined as a pass in which the animal moved three body lengths at a consistent speed and without turning (Basso et al. 2006). Separate scores were given for the left and right hindlimbs to assess if symptoms were bilateral or unilateral.

**Table 1:** VPS scoring criteria

Score	Description	Signs
0	Normal	Normal, weight-bearing, plantar stepping^a^ with tail up during walking passes^b^
1	Onset of symptoms	Weight-bearing, plantar stepping with mild rotation
	Tail position:	May be down or not fully up
	Miss-step:	Foot rotated on take-off or landing
	Weight bearing:	Wobble is present indicating weakness
2	Mild paresis	Mild miss-steps (but able to bear weight)
	Tail position:	Down
	Miss-step:	Mild, toe curling/dragging on ground foot slightly skids medially or laterally
	Weight bearing:	A limp may be present indicating weakness
	Joint movement:	May appear stiffer
3	Moderate paresis	Moderate miss-steps (but able to bear weight)
	Miss-step:	Obvious foot curling/dragging on ground foot obviously skids medially or laterally
	Weight bearing:	Limb is obviously weak
	Joint movement:	May appear stiffer
4	Severe paresis	Severe miss-steps (not bearing much weight)
	Miss-step:	Limb mostly drags behind, medially or laterally
	Weight bearing:	Not much, but limb still used to aid forward motion
	Joint movement:	Obviously decreased
5	Paralysis	No weight-bearing steps, slight joint movement
	Miss-step:	No stepping, limb only drags
	Weight bearing:	None
	Joint movement:	Slight
6	Complete paralysis	No weight-bearing steps, no joint movement
	Joint movement:	None

^a^Plantar stepping: Paw is placed flat on ground during stepping. It does not curl or skid to one side, and the toes/feet do not curl or drag at any point

^b^Walking pass: Animal moves three body lengths at a consistent speed and without turning

### Seizure-like activity score

Seizure-like activity noted during viral paresis scale (VPS) assessments was recorded as mild if the animal had mild shakes or tremors or severe if the animal was violently seizing in the cage.

### Histological analyses

Mice were perfused transcardially with phosphate-buffered saline (PBS) followed by 4% paraformaldehyde. The brain, kidney, liver, pancreas, hindlimbs, and lumbosacral spinal column were removed and post-fixed in the same fixative overnight, rocking at 4 °C. Tissues were rinsed twice in PBS, and the lumbosacral spinal cord, sciatic nerves, and gastrocnemius muscles were isolated. All tissues except the gastrocnemius muscle on the right were cryoprotected in 30% sucrose in PBS for 2–3 days at 4 °C, embedded in OCT compound (Ted Pella), and frozen with a dry ice/ethanol bath. Five sets of adjacent sections were cut on a cryostat at 25 μm, mounted on slides, and stored at − 20 °C until ready for further processing. For immunohistofluorescent staining, sections were encircled with a hydrophobic barrier pen (ImmEdge, Vector Labs), rinsed with PBS, and blocked with 10% normal serum and 1% Triton X-100 in PBS for 1 h. Primary antibodies were diluted in blocking solution as shown in Table 2 and applied to sections for incubation overnight at room temperature. Secondary antibodies conjugated to Alexa-488, Alexa-568, or Alexa-647 (from Invitrogen or Jackson ImmunoResearch) were diluted to 10 μg/mL in blocking solution. Sections were rinsed three times in PBS, incubated with secondary antibody solution for 2 h at room temperature, rinsed three times in PBS, incubated with Hoechst 33342 (Invitrogen, 1/2000 in PBS with 0.05% Triton X-100), and rinsed twice in PBS. Coverslips were mounted with Fluoromount G (Southern Biotech). For CD3 labeling, the amount of Triton X-100 was decreased to 0.5% in the blocking solution and 0.2% in the antibody diluent.

**Table 2:** Primary antibodies^a^

Antibody	Antibody type	Company, Catalog #	Dilution
ChAT	Goat pAb	Millipore, AB144P	1/100
ZIKV	Rabbit pAb	IBT, 0308-001	1/500
iba1	Goat pAb	Abcam, ab5076	1/200
GFAP	Rat IgG2a-kappa mAb	Invitrogen, 13-0300	1/500
CD3	Rabbit mAb	Abcam, ab16669	1/100
NF-H	Chick pAb	Aves Labs, NFH	1/200
MBP	Rat IgG2a mAb	Abcam, ab7349	1/200
Ly6G	Rat IgG2b mAb	Abcam, ab25377	1/500
α-Bungarotoxin-TMR		Biotium, 00012/00014	1/1000

*pAb* polyclonal antibody, *mAb* monoclonal antibody

^a^Summary of primary antibodies and dilutions used

Gastrocnemius muscles from the right side were sectioned longitudinally at 200 μm on a Vibratome (3000 plus, Vibratome Co.), collected in PBS, and stored at 4 °C. Ten to 12 sections were obtained for each muscle, and the third or fourth and seventh or eighth sections were chosen for neuromuscular junction (NMJ) and neurofilament (NF-H) staining as described in Itoh et al. (2011). Briefly, sections were incubated free floating in 0.1 M glycine in PBS, pH 7.3 for 30 min; blocked with 5% donkey serum (Jackson ImmunoResearch Laboratories), 0.5% Triton X-100, and 1% BSA in PBS containing tetramethylrhodamine (TMR)-conjugated alpha-bungarotoxin (α-btx) for 1 h; permeablized with 100% methanol at − 20 °C for 7 min; rinsed with PBS with 0.5% Triton X-100 (PBST); and incubated with primary antibody diluted in 1% BSA and 0.3% Triton X-100 in PBS at 4 °C for 48 h. After rinsing in PBST three times, sections were incubated at room temperature for 2 h with secondary antibody (anti-chicken conjugated to Alexa Fluor® 488, 1/100, Jackson ImmunoResearch Laboratories) diluted in PBS. After rinsing in PBST three times, sections were dehydrated through a methanol series (15 min each in 20, 50, 70, 100%), cleared with benzyl alcohol/benzyl benzoate (1:2) (Zukor et al. 2010), and stored at 4 °C until ready to image.

### Imaging and image processing

Fluorescent images were obtained at ×10 or ×20 with a laser scanning confocal microscope (Zeiss, LSM710) equipped with 405, 488, 561, and 633 laser lines. For images taken for pixel-based quantification, identical settings were used for all images in a set. For images chosen for publication, distracting artifacts were removed in ImageJ (Schneider et al. 2012) and levels were adjusted in Photoshop to maximize the signal-to-noise ratio so that relevant features could be seen more clearly. For images chosen to highlight pixel-based quantification, sham and ZIKV group images were adjusted identically to enable equitable comparison.

### Image quantification

ImageJ or FIJI was used for image quantification (Schneider et al. 2012). The cell counter plugin was used for manual cell counts. For pixel-based quantification of a given antibody signal, the area to be measured was defined by a region of interest (ROI); then, thresholds of single-channel images were adjusted to select pixels with signal above noise (positive pixels). Thresholds of all images in a set were adjusted identically for equitable comparison. Then, positive pixels within the whole area ROI were measured to obtain the area occupied by positive pixels. The area of positive pixels was divided by the whole area of the main ROI to determine what proportion of the ROI was positive for the antibody. For co-localization analysis, pixels that were positive for two antibody signals were measured.

**Fig. 4:**
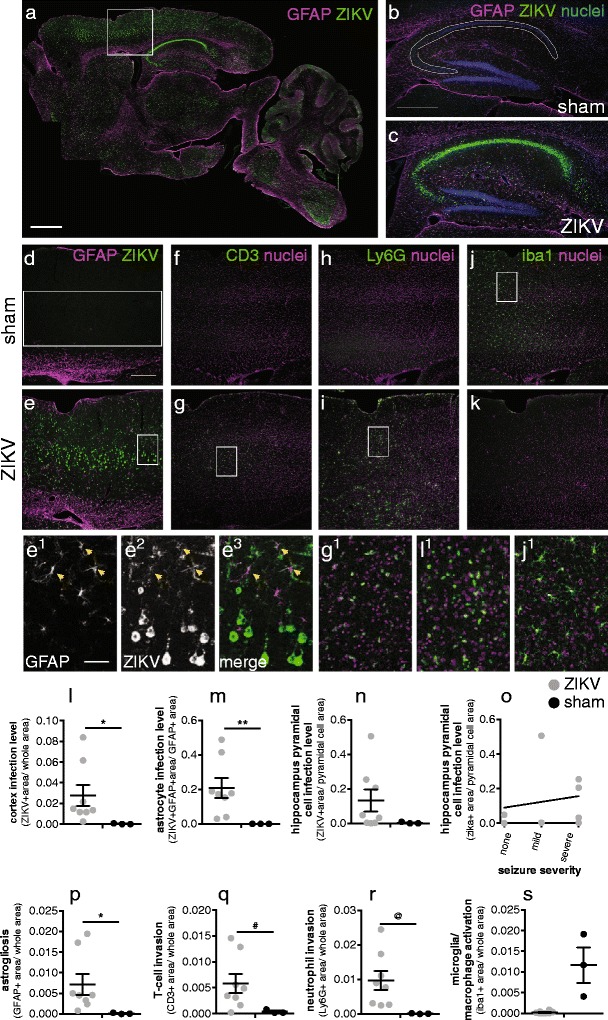
ZIKV can broadly infect the brain of adult, AG129 mice. (A) Sagittal section of the brain of the animal having the most pervasive CNS ZIKV infection, labeled with antibodies against GFAP (magenta) and ZIKV (green). (B, C) Examples of ZIKV infection (green) in the hippocampus of sham (B) and ZIKV (C, close-up of box in A) infected animals. Outlined region in B shows the region that was quantified in N. (D–K) Examples of sham (D, F, H, J) and ZIKV (E, G, I, K) infected motor cortex labeled with antibodies against GFAP (astrocytes, magenta) and ZIKV (green) (D, E), CD3 (T cells, green) (F, G), Ly6G (neutrophils, green) (H, I), and iba1 (microglia/macrophages, green) (J, K). Box in D shows the location and size of the region that was quantified in L, M, P–S. (E^1^–E^3^) Close-up of infected astrocytes in boxed area of E showing the GFAP (E^1^) and ZIKV (E^2^) channels separately and merged (E^3^). Arrowheads mark ZIKV+, GFAP+ astrocytes. (G^1^) Close-up of T cells in boxed area of G. (I^1^) Close-up of neutrophils in boxed area of I. (J^1^) Close-up of resting microglia in boxed area of J. (L, M, P–S) Quantification of signal in a region of motor cortex containing the layer V pyramidal neurons (corresponding to the boxed region shown in D). Each dot represents one animal. Bars indicate mean and SEM of each group. *p = 0.0315; **p = 0.0088; ^#^*p* = 0.0221; ^@^*p* = 0.0102. (N) Quantification of signal in the pyramidal cell layer of the hippocampus (corresponding to the outlined region shown in B). (O) Correlation between ZIKV infection level in the hippocampus pyramidal cell layer and severity of seizure-like activity. Line represents linear regression analysis. Scale bars = 1 mm (A), 500 μm (B, C same scale), 250 μm (D–K same scale), 50 μm (E^1^–E, G^1^, I^1^, J^1^ same scale)

Sagittal sections of the brain containing motor cortex were identified based on the morphology of the lateral ventricle about 1 mm lateral of bregma (Paxinos and Franklin 2004). A 1417 × 553 μm^2^ region containing the pyramidal cell layer of the motor cortex, in a region of the motor cortex just dorsal to the lateral ventricle, was measured for pixel-based quantifications (see box in Fig. 4 (D) for location of the region measured). Two sections per animal were measured for each parameter. For analysis of ZIKV in the hippocampus, one section per animal was measured, and the area measured corresponded to pyramidal cell layer (see Fig. 4 (B) for location of region measured).

**Fig. 5:**
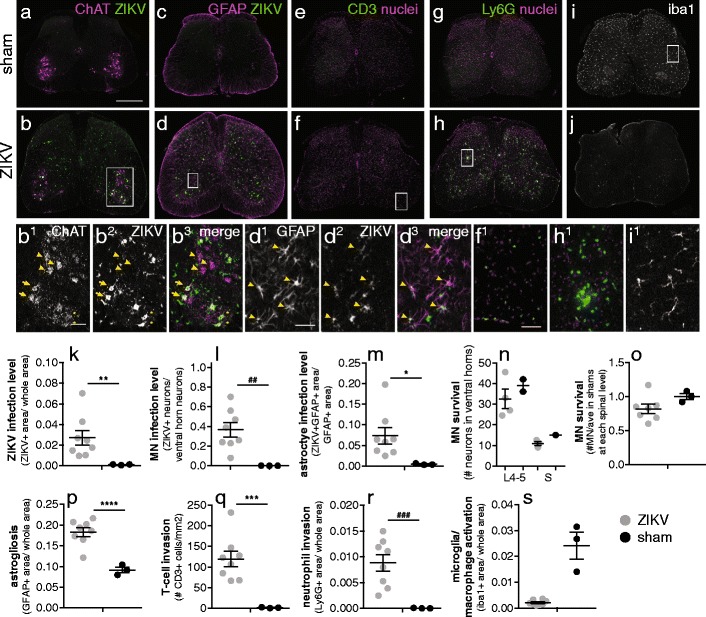
ZIKV heavily and consistently infects the lumbosacral spinal cord of adult, AG129 mice. (A–J) Examples of sham (A, C, E, D, I) and ZIKV (B, D, F, H, J) infected lumbar spinal cords (L4-5 level) labeled with antibodies against ChAT (motor neurons, magenta) and ZIKV (green) (A, B), GFAP (astrocytes, magenta) and ZIKV (green) (C, D), CD3 (T cells, green) (E, F), Ly6G (neutrophils, green) (G, H), and iba1 (microglia/macrophages, white) (I, J). (B^1^–B^3^) Close-up of infected motor neurons in boxed area of B showing the ChAT (B^1^) and ZIKV (B^2^) channels separately and merged (B^3^). Arrowheads mark ZIKV−, ChAT+ neurons; arrows mark ZIKV+, ChAT+ neurons, and asterisks mark ZIKV+ neurons with low levels of ChAT. (D^1^–D^3^) Close-up of infected astrocytes in boxed area of D showing the GFAP (D^1^) and ZIKV (D^2^) channels separately and merged (D^3^). Arrowheads mark ZIKV+, GFAP+ astrocytes. (F^1^) Close-up of T cells in boxed area of F. (H^1^) Close-up of neutrophils in boxed area of H. (I^1^) Close-up of resting microglia in boxed area of I. (K–S) Quantification of images. Each dot represents one animal. Bars indicate mean and SEM of each group. **p = 0.0088; ^##^*p* = 0.0014; *p = 0.0104; ****p < 0.0001; ***p = 0.0004; ^###^*p* = 0010. Scale bars = 500 μm (A–J same scale), 100 μm (B^1^–B^3^ same scale), 50 μm (D^1^–D^3^ and F^1^–I^1^ same scale)

Spinal cord levels were identified using the mouse spinal cord atlas (Watson et al. 2009) and the location and morphology of clusters of choline acetyltransferase (ChAT) positive neurons. Because motor neurons for the gastrocnemius muscle are located at the L4-L5 level (McHanwell and Biscoe 1981), sections from this level were chosen for analysis when possible. For ventral motor neuron, astrocyte, and ZIKV infection level analyses, two sections per animal were analyzed. ZIKV−, ChAT+ and ZIKV+, ChAT+ neurons in the ventral horns were counted manually using the cell counter in ImageJ. Because there were occasionally ZIKV+ cells in the ventral horns with neuronal morphology that had low or undetectable ChAT levels (Fig. 5 (B^1^–B^3^)), these cells were also counted as ventral motor neurons. For analysis of Ly6G (neutrophils), CD3 (T cells), and iba1 (microglia/macrophages) signals, one section per animal was analyzed.

**Fig. 6:**
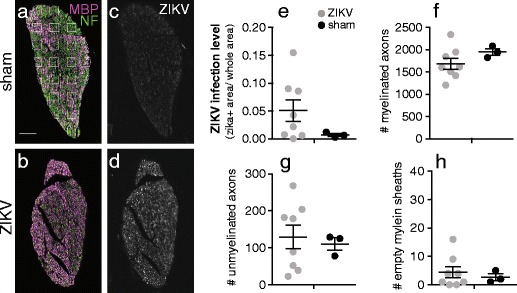
ZIKV can infect sciatic nerves of adult, AG129 mice, but axons and myelin appear healthy. **a**–**d** Examples of sham (**a**, **c**) and ZIKV (**b**, **d**) infected sciatic nerves labeled with antibodies against MBP (myelin, magenta) and NF (axons, green) (**a**, **b**) and ZIKV (white) (**c**, **d**). Grid in **a** shows the counting grid used for quantification in **f–h***.* **e–h** Quantification of ZIKV signal (**e**) and number of myelinated axons (**f**), unmyelinated axons (**g**), and empty myelin sheaths (**h**) in the whole cross section of nerve. Each dot represents one animal. Bars indicate mean and SEM of each group. Scale bar = 50 μm (**a–d** same scale)

For the nerve cross sections, a stereological counting grid containing 25 × 25 μm^2^ boxes where each box was spaced 25 μm from the boxes above and below it was placed over the nerve to obtain counts from a sample of the nerve, representing approximately one fourth of the total area. An ROI was placed around the whole nerve, and the counting grid was cut to eliminate all areas of the grid outside of the nerve (see Fig. 6a). Stereological counting rules were used to count the number of myelinated axons, unmyelinated axons, and empty myelin sheaths within the boxes of the cut grid. Images were converted to composite images, so the neurofilament and myelin basic protein (MBP) channels could be toggled on and off during counting. All counts were done by a researcher who was blind to the status of each animal. The area of the cut grid as well as the area of the whole ROI were calculated and used to extrapolate the number of myelinated axons, unmyelinated axons, and empty myelin sheaths within the whole area. Three sections per animal were analyzed.

For NMJ analysis, two sections per animal were analyzed. Confocal z-stacks (~ 1-μm step size) of two optimal fields of view containing NMJ endplates (α-btx+) and nerve fibers (NF-H+) were analyzed per section for a total of four fields of view per animal. The number of endplates and the number of innervated endplates (co-localized with some neurofilament signal) were counted manually with the cell counter in ImageJ. Images were converted to composite images, so the NF-H and α-btx channels could be toggled on and off during counting. Total numbers of innervated NMJs (NF-H+, α-btx+) and all NMJs (α-btx+) from all four fields analyzed were used to calculate the percentage of NMJs that were innervated per animal.

### Gastrocnemius CMAP

CMAP measurements were performed by a researcher who was blind to the infection status and VPS score of each mouse. Animals were anesthetized with isoflurane and maintained at 37 °C body temperature with a rectal probe and heating pad, and their hindlimbs were shaved. Monopolar needle electrodes (Tai-Chi Brand, acupuncture needles) were inserted into the sciatic notch or epidural to the lumbosacral spinal cord (after surgical exposure) to stimulate the gastrocnemius muscle with 0.1-ms pulses of current using a stimulus isolator (WPI Isostim A320). For lumbosacral spinal cord stimulation, monopolar needle electrodes (BIOPAC Systems, EL452) were used, and the anode was inserted between the T13 and L1 vertebrae, and the cathode was inserted between the L1 and L2 vertebrae (Basoglu et al. 2013; Harrison et al. 2013). No laminectomy was necessary. Muscle responses were recorded with 4-mm-diameter shielded Ag/AgCl surface recording electrodes (EL254S, Biopac system) filled with electrode gel. Electrodes were placed on the skin at the belly of the muscle and at the anterior aspect of the ankle and connected to a differential amplifier (DAM 50, WPI) with a gain of ×100 and filtered with a 300-1K Hz band pass filter. Data were acquired at a 20 K/s sampling rate with Powerlab 4/25 and LabChart 8 software (ADInstruments). Responses to five pulses at 1 Hz were averaged, and the current was increased incrementally until a maximum amplitude was reached. Maximal CMAP amplitudes were measured from peak to peak of the M wave.

For experiments with α-btx, uninfected, male AG129 mice, about 2.5 months of age, were used. After finding the stimulation current that gave the maximal CMAP response, 50 μl of 12.5, 6.25, or 3.13 μM α-btx (Biotium, Inc., #00010-1) was injected into the gastrocnemius muscle (with the gel pads kept in place) with a 26G needle (Nakanishi et al. 2005). CMAP responses after maximal stimulation current were recorded at intervals for up to 1 h after α-btx injection. Dilutions of α-btx were made with saline, and thus, a 50 μl saline injection was used in the control.

### Statistics

Data were graphed and analyzed with Prism (GraphPad Software, Inc.) for statistical significance using *t* tests or two-way ANOVAs with post hoc *t* tests. Linear regression was used for correlation analyses.

## Results

### Experiment no. 1 disease and behavioral motor deficits

ZIKV-infected animals began losing weight by 7 DPI and started succumbing to the disease by 11 DPI (Fig. 1). By 16 DPI, all ZIKV-infected animals had died or been humanely euthanized.

The onset of hindlimb motor deficits in ZIKV-infected mice ranged from 8 to 13 DPI (Fig. 2a, b). Initial symptoms included tail weakness (tail not in the up position during walking) and subtle hindlimb lateral skidding and weakness (as indicated by a limp or wobble). Disease progressed quickly after the onset of motor deficits and morbidity occurred within 2–3 days. The severity of motor deficits generally increased rapidly, with VPS scores reaching as high as 5 or 6 before death (hindlimb dragging with little to no joint movement). Mice were grouped into early (8–11 DPI) and late (12–13 DPI) onset groups in Fig. 2c, d so that scores from later onset mice would not mask the progression of disease in the early onset mice. Deficits were more severe on the right side compared with the left, corresponding to the side of virus inoculation. Forelimbs were rarely affected.

Seizure-like activity was also observed in ZIKV-infected mice (Fig. 3). Six of 10 ZIKV-infected mice had some seizure-like activity, and 4 mice were scored as severe (Fig. 3a). Seizure-like activity did not correlate with VPS score (Fig. 3b). Half of the mice in which severe seizure-like activity was noted had no to only subtle hindlimb deficits after seizure activity had subsided. Additionally, there were mice with complete paralysis (VPS score of 6) that were never observed to have seizure-like activity. Two other disease phenotypes, walking in circles and extreme mobile activity (not shown), were also observed (data not shown).

Interestingly, the severity of hindlimb paralysis and seizure-like activity did not correlate well with changes in body weight (Supplementary Fig. S1), suggesting that mechanisms affecting neuroinvasion might operate independently from those affecting systemic infection and body weight changes.

### Experiment no. 1 immunohistofluorescence

Initial histopathologic analysis of our relatively thick, frozen sections with hematoxylin and eosin staining was considered to be preliminary. Nevertheless, the following preliminary results were evidence of mild to moderate, multifocal encephalitis in the brain; moderate to severe, multifocal myelitis in the spinal cord; and no lesions in the gastrocnemius muscle or sciatic nerve (data not shown). To further identify anatomical features that might be associated with motor deficits, we performed immunohistofluorescent analyses of the motor cortex, lumbosacral spinal cord, sciatic nerve, and gastrocnemius muscle. The kidney, liver, and pancreas were collected to assess systemic infection level.

### Motor cortex

Using a polyclonal antibody against ZIKV envelope glycoprotein, ZIKV immunoreactive (ir) was seen in all parts of the adult AG129 mouse brain, including motor cortex, cerebellum, hippocampus, and brainstem (Fig. 4 (A)). In the motor cortex, ZIKV infects both neurons and astrocytes (arrows in Fig. 4 (E^1^–E^3^)), and many of the neurons have the morphology and location of layer V pyramidal neurons which are upper motor neurons that project to the spinal cord (Fig. 4 (E and E^1^–E^3^)). Quantification of a region of the motor cortex containing layer V (shown in box in Fig. 4 (D)) revealed that 1–2% and up to 6–8% of the area were ZIKV ir in ZIKV-infected mice (Fig. 4 (L)). Additionally, 20–50% of astrocytes in this area were ZIKV ir (Fig. 4 (M)), as measured by pixel-based quantification.

There were also significant increases in astrogliosis (Fig. 4 (E, E^1^–E^3^, and P)), T cell infiltration (Fig. 4 (G, G^1^, and Q)), and neutrophil infiltration (Fig. 4 (I, I^1^, and R)) in the ZIKV-infected motor cortex compared to sham infected (Fig. 4 (D, F, and H)). Interestingly, microglia and macrophages were virtually undetectable in the ZIKV-infected AG129 mouse brain (Fig. 4 (K and S)), though resting microglia were apparent in sham-infected brains (Fig. 4 (J and J^1^).

### Hippocampus

The pyramidal cell layer of the hippocampus was also strikingly ZIKV ir in many ZIKV-infected mice (Fig. 4 (C)). Quantification of this region (outlined in Fig. 4 (B)) demonstrated that 20–50% of this layer was ZIKV ir in three of eight mice (Fig. 4 (N)). Because of the association between the hippocampus and seizure activity, the severity of seizure-like activity was plotted against hippocampus pyramidal cell layer ZIKV infection levels, but no correlation was found (Fig. 4 (O)). Likewise, no correlation was found between motor cortex infection level and seizure-like activity (data not shown).

### Lumbosacral spinal cord

ZIKV ir was also abundant in the lumbosacral spinal cord which contains lower motor neurons that innervate the hindlimbs (Fig. 5 (A–D)). Overall, 1–7% of the cross-sectional area was ZIKV ir (Fig. 5 (K)). As with the brain, astrocytes (arrowheads in Fig. 5 (D^1^–D^3^ and M)) and neurons were infected. Using a ChAT antibody to label ventral motor neurons, many were found to be ZIKV ir (arrows in Fig. 5 (B^1^–B^3^)) and a few ventral cells with clear neuronal morphology were strongly ZIKV ir, but only weakly ChAT ir (asterisks in Fig. 5 (B^1^–B^3^)). As many as 20–70% of ventral motor neurons were ZIKV ir (Fig. 5 (L)), which suggests that ZIKV can infect and replicate in lower motor neurons in the spinal cord. The number of motor neurons at the L4-L5 and S levels was also diminished in many ZIKV-infected mice (Fig. 5 (N and O)), but the difference from sham-infected mice did not reach statistical significance, likely because the disease progressed so rapidly.

As in the brain, astrogliosis (Fig. 5 (C, D, and P)), T cell infiltration (Fig. 5 (E, F, F^1^, and Q), and neutrophil infiltration (Fig. 5 (G, H, H^1^, and R) were markedly increased in ZIKV-infected mice compared to sham-injected mice. Conversely, microglia and macrophages were markedly reduced or absent (Fig. 5 (J and S)) in these ZIKV-infected AG129 mice, as observed in the brain, though resting microglia were evident in sham-infected spinal cords (Fig. 5 (I and I^1^).

### Sciatic nerve

ZIKV ir reactivity was detected in cross sections of the sciatic nerve (Fig. 6c–e); however, this did not appear to have a statistically significant effect on the number of myelinated axons (Fig. 6a, b, f), unmyelinated axons (Fig. 6g), or empty myelin sheaths (Fig. 6h).

### Gastrocnemius muscle, kidney, liver, and pancreas

**Fig. 7:**
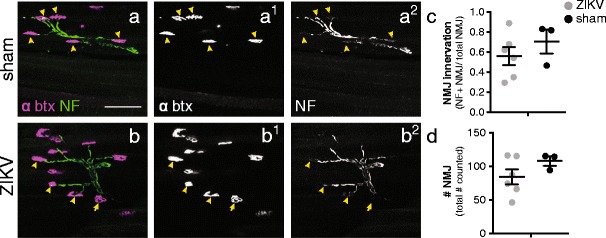
NMJs appear normal after ZIKV infection of adult, AG129 mice. (A, B) Examples of sham (A) and ZIKV (B) infected gastrocnemius muscle labeled with TMR-α-btx (NMJ endplate, magenta) and an antibody against NF (axons, green). (A^1^, B^1^) α-btx channel alone. (A^2^, B^2^) NF channel alone. Arrowheads mark innervated NMJs (α-btx+ and NF+). (C, D) Quantification of percent of NMJs that are innervated (C) and total number of NMJs counted (D). Each dot represents one animal. Bars indicate mean and SEM of each group. Scale bar = 50 μm (A, B same scale)

ZIKV ir and neutrophil infiltration were not detected in the gastrocnemius muscles of ZIKV-infected mice (data not shown). Infection of lower motor neurons in the spinal cord and their axons in the sciatic nerve did not appear to lead to a statistically significant loss of NMJ number or innervation (Fig. 7). No ZIKV ir was detected in the kidney, liver, or pancreas of ZIKV-infected mice (data not shown).

### Correlations

**Fig. 8:**
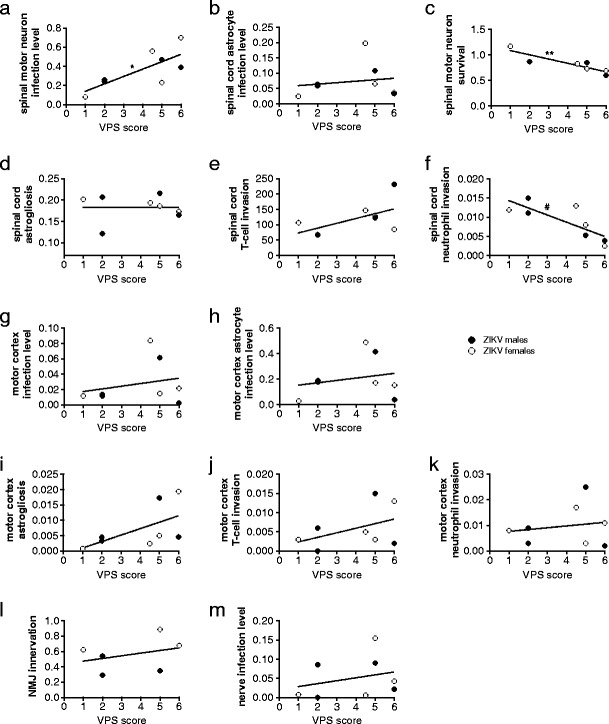
Motor deficits correlate with spinal motor neuron infection level, spinal motor neuron survival, and spinal cord neutrophil infiltration. **a–m** Image quantification parameters shown in Figs. 4, 5, 6, and 7 graphed against the highest VPS score seen in the animal. Each dot represents one ZIKV-infected animal (gray = female, black = male). Lines represent linear regression analysis. *p = 0.0317; **p = 0.0071; ^#^*p* = 0.0164

To determine which anatomical features might be correlated with hindlimb motor deficits, we used our data to make preliminary correlations between histological parameters of the spinal cord (Fig. 8a–f), motor cortex (Fig. 8g–k), NMJ (Fig. 8l), and sciatic nerve (Fig. 8m) and VPS scores. Statistically significant correlations were only found with spinal cord parameters. Increased numbers of ZIKV-infected ventral motor neurons in the lumbosacral spinal cord are associated with higher VPS scores (Fig. 8a, *p* < 0.05), and higher VPS scores are associated with decreased survival of spinal motor neurons (Fig. 8c, *p* < 0.001). These data suggest that ZIKV infection of lower motor neurons and lower motor death adversely affects hindlimb function. Interestingly, the level of neutrophil infiltration was inversely proportional to VPS score (Fig. 8f, *p* < 0.05), which suggests that the presence of neutrophils might mitigate motor deficits. To get a preliminary indication of how neutrophil invasion and spinal motor neuron viral infection are related to spinal motor neuron survival (as opposed to VPS score), correlation graphs with these parameters were prepared as well and similar trends were observed (Supplemental Fig. S2). VPS scores did not appear to correlate with virus levels in the motor cortex (Fig. 8g, h) or with sciatic nerve infection levels (data not shown).

Overall, these results suggest that hindlimb motor deficits in these AG129 mice during the acute phase of ZIKV infection are likely caused primarily by spinal cord myelitis, though upper motor neuron disease and peripheral neuropathy may contribute.

### Experiment no. 2 disease and behavioral motor deficits

Experiment no. 2 reproduced the disease and motor phenotypes seen in experiment no. 1. ZIKV-infected animals began losing weight by 7 DPI and started succumbing to the disease by 11 DPI (Supplemental Fig. S3). All ZIKV-infected mice had died or been humanely euthanized by 14 instead of 16 DPI. VPS assessments were done less frequently, but symptoms appeared at a similar timing and progressed at a similar rate (Supplemental Fig. S4).

### Experiment no. 2 electrophysiological assessment

When mice in experiment no. 2 had VPS scores ≥ 3, gastrocnemius muscle CMAPs were recorded in response to stimulation at the sciatic notch, followed by stimulation of the lumbosacral spinal cord to further delineate the cause of the hindlimb motor deficits. We reasoned that since motor deficits progress quickly after symptom onset and many motor neurons, though infected, have not died by the time severe deficits occur, axons and NMJs may still be healthy even if the neuronal cell body is sick. Thus, CMAP amplitudes may be normal in response to sciatic notch stimulation, but decreased upon lumbosacral spinal cord stimulation. Responses to stimulation at these two sites, therefore, might distinguish between myositis/neuritis and myelitis.

**Fig. 9:**
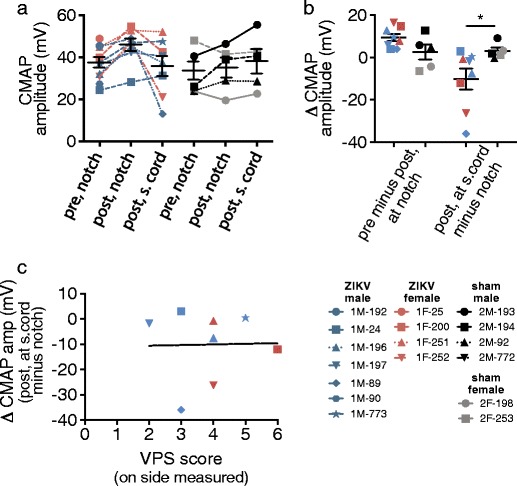
Relative gastrocnemius muscle CMAP amplitudes in response to lumbosacral spinal cord stimulation are reduced after ZIKV infection. **a** Gastrocnemius muscle CMAP amplitude pre-infection in response to sciatic notch stimulation (pre, notch); post-infection in response to sciatic notch stimulation (post, notch); post-infection in response to lumbosacral spinal cord stimulation (post, s. cord). Post-infection measurements were obtained when VPS score ≥ 3. **b** Difference between CMAP amplitudes (also referred to as “relative” CMAP amplitudes). For “pre minus post, at notch,” the pre-infection value was subtracted from the post-infection value in response to sciatic notch stimulation. For “post, s. cord minus notch,” the post-infection value in response to sciatic notch stimulation was subtracted from the value in response to lumbosacral spinal cord stimulation. *p = 0.0355*.* **c** Relative CMAP amplitudes in response to lumbosacral spinal cord stimulation (post, s. cord minus notch in **b**) plotted against the VPS score on the measured side. Each symbol represents one animal. Bars indicate mean and SEM of each group. Lines in **a** and **b** connect measurements from same animal (symbols of individual animals are connected). Line in **c** represents linear regression analysis

Because CMAP measurement in response to lumbar spinal cord stimulation was an invasive procedure, pre-infection baselines were only obtained in response to sciatic notch stimulation. Figure 9a shows the CMAP amplitudes for each animal pre-infection and post-infection in response to stimulation at the two sites. There are no statistically significant differences between groups when these “absolute” values are used. The use of relative values, however, are more compelling, and may be necessary because individual differences in the thickness and composition of tissue between the surface of the skin and the muscle can affect CMAP amplitudes (Nordander et al. 2003). When post-infection CMAP amplitude was expressed relative to pre-infection amplitude (both in response to sciatic notch stimulation), there was no statistically significant difference between the ZIKV-infected and sham-infected groups (Fig. 9b, “pre minus post, at notch”), suggesting that the axons and NMJs are healthy after infection and motor deficits are not due to myositis or neuritis. When post-infection CMAP amplitude in response to stimulation of the spinal cord was expressed relative to the amplitude in response to stimulation at the sciatic notch, however, there was a statistically significant relative decrease in amplitude in the ZIKV-infected group (Fig. 9b, “post, at s. cord minus notch,” *p* < 0.05). This suggests that, after infection and at the time motor deficits are observed, the axons and NMJs are healthy enough to generate a normal CMAP response when axons are stimulated at the sciatic notch, but the neuronal cell bodies in the spinal cord are impaired and less able to generate normal CMAP responses when stimulated at the lumbosacral spinal cord. This indicates that myelitis, rather than myositis or neuritis, contributes to the motor deficits. The fact that VPS score did not correlate with the relative CMAP amplitude in response to spinal cord stimulation (Fig. 9c), however, suggests that other factors also contribute to hindlimb motor deficits.

**Fig. 10:**
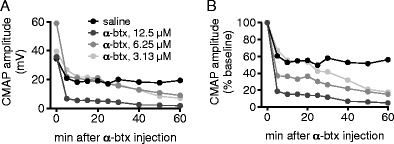
Gastrocnemius muscle activity contributes to CMAPs. **a** Gastrocnemius CMAP amplitudes from uninfected AG129 male mice before and after injections of α-btx. **b** CMAP amplitudes expressed as a percentage of baseline

To validate that the CMAP indeed reflected muscle activity, and not just nerve activity, an inhibitor of the NMJ nicotinic acetylcholine receptor, α-btx, was injected into the gastrocnemius muscles of uninfected adult AG129 mice, and CMAPs were recorded for up to 1 h after injection. CMAP amplitudes decreased in a dose-dependent manner over the course of the 1 h after α-btx injection, whereas CMAP amplitude in the saline-injected animal was relatively stable (Fig. 10). These data suggest that the CMAP reflects mostly muscle rather than nerve activity, and thus, CMAP measured the health status of all structures between the point of stimulation and the muscle (including the nerve and NMJ).

## Discussion

This study demonstrates that a contemporary strain of ZIKV (PRVABC59) can widely infect astrocytes and neurons in the brain and spinal cord of adult AG129 mice and cause rapidly progressing hindlimb paralysis, as well as severe seizure activity, during the acute phase of disease. Motor deficits in these circumstances appear to be primarily due to myelitis and possibly encephalitis as opposed to a peripheral neuropathy or GBS-like syndrome. This is an important new finding because the most severe histopathologies previously reported in AG129 mice were in the brain and muscle (Aliota et al. 2016). The finding that myelitis is likely the primary cause of ZIKV-induced paralysis is also in alignment with what has been seen with other flavivirus-induced motor deficits (Sejvar et al. 2003; Solomon et al. 1998). This conclusion is also supported by our data suggesting that muscle and peripheral nerve functions are normal. Electrophysiological CMAP amplitude measurements of sciatic nerve and sural muscles were normal in response to stimulation at the sciatic notch. Additionally, the anatomy of the sciatic nerve and gastrocnemius muscle, as revealed by neurofilament/MBP and neurofilament/NMJ staining, respectively, was minimally disrupted. In contrast, ZIKV ir was high, intense, and obvious in the brain and spinal cord. The severity of hindlimb motor deficits correlated with increased numbers of ZIKV-infected lumbosacral spinal motor neurons and decreased numbers of spinal motor neurons. Relative CMAP amplitudes upon stimulation at the lumbar spinal cord were also reduced when obvious motor deficits were present (VPS score ≥ 3). ZIKV infections in the brain and spinal cord were also associated with astrogliosis as well as T cell and neutrophil infiltration. Thus, acute ZIKV infection of adult AG129 mice may be a useful model for studying the mechanisms of ZIKV-induced myelitis (Anaya et al. 2017; Dirlikov et al. 2016), encephalitis, and seizure activity.

### Myelitis

The results of this study may be clinically relevant, because there have been several reports of myelitis during recent ZIKV outbreaks. In the February 2016 outbreak in Guadeloupe, a 15-year-old girl developed hemiparalysis that was associated with detection of ZIKV in the cerebrospinal fluid. Magnetic resonance imaging revealed extensive lesions in the cervical and thoracic spinal cord (Mecharles et al. 2016). Investigators reporting on ZIKV-associated GBS in Puerto Rico noted 26 cases of neurologic disorders other than GBS, 2 of which were myelitis (Dirlikov et al. 2016). In a case-controlled study in Colombia from a national population-based surveillance system (Anaya et al. 2017), 29 patients with ZIKV-associated GBS were compared to matched controls. Thirteen of these patients were reported to have other neurological syndromes: three with myelitis, three with peripheral facial palsy, six with transverse myelitis, and one with thoraco-lumbosacral myelopathy.

The observation that ZIKV can infect and kill spinal motor neurons and that this correlates with hindlimb motor deficits is a unique finding of this report and may be clinically important. Pathology reports of human ZIKV infection are limited, but do reveal neuronophagia in the brain (Solomon et al. 2016), which suggests that ZIKV may be capable of eliciting neuron death in human neurological tissues as well as in the AG129 mouse. ZIKV infection has previously been reported in the spinal cords of AG129 mice (Julander et al. 2017; Zmurko et al. 2016), and ZIKV-induced histopathology or myelitis has been observed in IFNAR^−/−^ (Lazear et al. 2016) and Swiss Webster mice infected as newborns (Fernandes et al. 2017).

### Myelitis in other flavivirus infections

The ZIKV-induced motor deficits in AG129 mice are reminiscent of motor deficits caused by other flaviviruses, such as WNV, in immunocompetent mice and hamsters (Morrey et al. 2004; Morrey et al. 2008; Shrestha et al. 2003; Siddharthan et al. 2009; Xiao et al. 2001; Zukor et al. 2017). These flavivirus models also demonstrate virus infection and killing of spinal motor neurons together with mild to severe signs of motor impairment (Zukor et al. 2017).

### Encephalitis

The vast majority of adults infected with ZIKV do not develop severe neurological disease such as encephalitis, yet the AG129 mice of this study are immunodeficient and develop encephalitis and myelitis. As such, the AG129 mouse model is probably more relevant to infection of immunocompromised patients. Some clinical reports of infected immunocompromised patients describe severe neurological complications (Henry et al. 2017; Schwartzmann et al. 2017). For example, an immunosuppressed heart transplant patient developed acute neurological impairment and mental deterioration after ZIKV infection, which culminated in death. Autopsy revealed ZIKV infection of the brain and cerebral spinal fluid by RT-PCR, immunohistochemistry, and electron microscopy. The histopathology of the brain was consistent with meningoencephalitis (Schwartzmann et al. 2017).

The fact that relative CMAP amplitude after spinal cord stimulation did not statistically correlate with VPS score suggests that damage upstream of spinal motor neurons may contribute to the hindlimb deficits. For example, CMAP amplitude was not reduced in two infected mice with overt paralysis (VPS score 5–6, Fig. 9c). Paralysis in these animals could be caused by motor cortex, cerebellum, or brainstem dysfunction, as extensive infection was observed in these areas of the brain. While upper motor neuron disease is associated with rigid, rather than flaccid paralysis, we occasionally saw symptoms that could be interpreted as rigidity, such as walking with high haunches and extended/stiff limbs, but it was difficult to reliably assess this with the VPS test. Another possible explanation for the paralysis in animals with normal relative CMAP amplitudes is that spinal motor neuron infection damages dendrites and post-synaptic densities such that the neurons are not able to receive signals physiologically, but when stimulated externally with an electrode, the axon hillock and axon are healthy enough to propagate an action potential to the muscle.

### Seizures

The observation of seizure-like activity in infected AG129 mice in this study and in immunocompetent mice infected as neonates in another study (Manangeeswaran et al. 2016) may have clinical relevance since recent ZIKV infections have been associated with seizures in humans (Asadi-Pooya 2016; Pastula et al. 2016). Notably, the seizure-like activity in this study was not correlated with the VPS score, which suggests that the seizure activity was not simply a manifestation of motor deficits. To further investigate the relevance, seizures would need to be confirmed in ZIKV-infected mice with more detailed analyses.

### Peripheral neuropathy

Motor deficits seen in this study are not likely to be the result of peripheral neuropathy. While ZIKV ir was observed in the sciatic nerve, axon and myelin morphology did not appear to be altered. ZIKV also did not appear to alter the function of the sciatic nerve, which is supported by the observation that gastrocnemius CMAP amplitudes in response to sciatic notch stimulation were not affected. The observation of viral antigen in axons is consistent with prior findings that four families of viruses, including flaviviruses, can undergo axonal transport and spread in the nervous system (Samuel et al. 2007; Taylor and Enquist 2015; Wang et al. 2009), as well as the finding that ZIKV appears to be able to travel in axons (van den Pol et al. 2017). We were not able to determine what effect ZIKV might have on the development of peripheral neuropathies, such as GBS, in this study because the mice die during the acute infection and peripheral neuropathies tend to develop in chronic stages.

### Cellular immune responses

In the current study, inflammatory cellular responses, such as astrogliosis, neutrophil infiltration, and T cell infiltration, were present in the brain and spinal cord. The possible influence of each of these responses to disease severity, whether causative or protective, is explored below. Future work should comprehensively establish correlation with larger sample sizes and then determine which relationships are causative or protective, so that the cellular mechanisms underlying ZIKV-induced motor deficits can be understood.

### Neutrophils

Results from this study suggest that neutrophils play a role in mitigating or protecting ZIKV-infected AG129 mice from motor deficits, probably by enhancing immune responses that reduce viral load. Increased numbers of neutrophils, as detected by the Ly6G antibody, were inversely correlated with VPS score (*p* < 0.05). Studies with WNV demonstrate that neutrophils can play complex roles during infection. They appear to be protective early in WNV infection, but detrimental later. When neutrophils were depleted before WNV challenge in C3H/HeJ mice, mortality increased, but when they were depleted after WNV challenge, mortality decreased (Bai et al. 2010). Another level of complexity regarding the role of neutrophils involves mosquito bites. When neutrophils are depleted and inflammasome activity is blocked, the ability of mosquito bites to promote infection and inflammation is abrogated (Pingen et al. 2016). Therefore, the role of neutrophils in ZIKV-induced disease should be further investigated.

### Microglia and macrophages

The drastic reduction in iba1 ir (a marker for microglia and macrophages) in the brains and spinal cords of AG129 mice infected with ZIKV was a unique finding. It could mean that microglia and macrophages are present, but the iba1 marker is strongly downregulated, or it could mean that microglia are killed and macrophage infiltration is inhibited. After WNV infection in C57BL6 mice, microglia and macrophages are strongly activated (Zukor et al. 2017). They are also strongly activated in wild-type C57BL6 or 129S1/SvImJ mice infected with ZIKV intracerebrally at E14.5 (Shao et al. 2016). Thus, the decrease in iba1 ir after ZIKV infection in our study might be unique to the AG129 mouse strain and could indicate a role for microglia and macrophages in protecting against lethal ZIKV infection. For example, M1 macrophages elicit pro-inflammatory responses and enable phagocytosis and killing of pathogens, and M2 macrophages function in protective and homeostasis of the CNS (Kabba et al. 2018). A certain balance of these functions could be overall protective. To further elucidate immunological cellular responses, decreased iba1 ir cells and modulation of other immune cells should be validated with flow cytometric analysis in future studies.

### T cells

T cell infiltration was associated with ZIKV infection in the brain and spinal cord, though there was no correlation between the level of infiltration and the severity of motor deficits. This could indicate that T cells play both beneficial and detrimental roles (Ousman and Kubes 2012; Prinz and Priller 2017). For example, CD8(+) T cells contribute to resolution of WNV neurological infection by helping to clear WNV from neurons (McCandless et al. 2008; Shrestha et al. 2012). T cells also play beneficial roles in La Crosse virus infection (Winkler et al. 2017). T cells, however, are the cause of fatal meningoencephalitis after Tacaribe arenavirus infection (Ireland et al. 2017). Future studies will delineate the role of T cells in the development of motor deficits in ZIKV-infected AG129 mice.

### Astrocytes

Astrogliosis in the spinal cord was strongly associated with ZIKV infection, and many astrocytes were infected by the virus. As astrocytes regulate synapse formation, function, and elimination (Chung et al. 2015) and control glutamate excitotoxicity (Murphy-Royal et al. 2017), this likely had a significant effect on motor neuron health and function. In a model of human coronavirus, expression of the primary glutamate transporter in the adult CNS, GLT-1, was decreased in astrocytes. This was associated with flaccid hindlimb paralysis even in the absence of significant neuronal death. Blockade of 2-amino-3-(5-methyl-3-oxo-1,2-oxazol-4-yl) propranoic acid (AMPA) receptors with a non-competitive antagonist improved hindlimb function (Brison et al. 2011). In mouse models of experimental autoimmune encephalomyelitis, astrocytes are implicated in the temporary stripping of excitatory synapses from motor neurons in the spinal cord, leading to severe flaccid paralysis in the absence of neuronal cell death (Blakely et al. 2015; Marques et al. 2006). Astrogliosis may not have correlated with VPS scores in our studies because it may precede and be required for the onset of motor deficits (Blakely et al. 2015).

### Susceptibility to disease

In two independent experiments, female mice were found to die slightly earlier than male mice, though this was not statistically significant. Other ZIKV studies with AG129 mice at our institute, however, suggest that males and females are equally susceptible (data not shown). Additionally, experiment no. 1 appeared to have two distinct groups of mice with an early versus late onset of symptoms, though this separation was not distinct in experiment no. 2. We interpret these trends (greater female susceptibility and two phases of disease onset) to be the result of stochastics and small sample sizes. The appearance of two onset groups in experiment no. 1 occurred by chance because we did not sample mice with an intermediate onset of symptoms.

## Conclusion

The unique contributions of this study are that acute ZIKV infection of adult AG129 mice causes quantifiable, rapidly progressing hindlimb motor deficits that often culminates in full paralysis and that these deficits are likely due to myelitis and perhaps encephalitis rather than peripheral neuropathy or myositis. Extensive histopathology and infection occur in the spinal cord and brain, but not in the sciatic nerve or muscle. The severity of motor deficits is only correlated with infection and survival levels of lumbosacral spinal motor neurons. Moreover, gastrocnemius CMAP amplitudes upon electrical stimulation of the lumbosacral spinal cord were impaired, but not upon stimulation of the sciatic nerve. These data should guide approaches for understanding ZIKV-induced acute myelitis or encephalitis in adults.

## Electronic supplementary material

Supplementary Figure S1 Weight loss does not appear to correlate well with the severity of hindlimb deficits (**A**) or seizure-like activity (**B**). Each dot represents one ZIKV-infected animal (gray = female, black = male). Lines represent linear regression analysis. Slopes were not significantly different from zero. (GIF 18 kb)

High resolution image (EPS 733 kb)

Supplementary Figure S2 Spinal motor neuron survival trends towards an association with a lower level of spinal motor neuron infection (**A**) and increased neutrophil invasion (**B**). Each dot represents one ZIKV-infected animal (gray = female, black = male). Lines represent linear regression analysis. Slopes were not significantly different from zero. (GIF 20 kb)

High resolution image (EPS 760 kb)

Supplementary Figure S3 Weight loss and survival curves for AG129 adult mice infected with ZIKV in Experiment #2. ***A***, Body weight expressed in terms of percent of starting weight after ZIKV or sham infection. ***B***, Percentage of each group surviving at each day after ZIKV or sham infection. All sham-infected animals (male and female) are represented in the sham-group. (GIF 37 kb)

High resolution image (EPS 747 kb)

Supplementary Figure S4 Moderate paresis to severe paralysis before death in ZIKV-infected AG129 mice was reproducible. ***A, B,*** VPS scores from Experiment #2 of individual mice on the right (**A**) and left sides (**B**). ***C, D,*** Mean VPS scores of ZIKV-infected mice that died early (*n* = 2) or late (*n* = 9) compared to sham infected mice (*n* = 6) on the right (**C**) and left sides (**D**). Error bars represent SEM. (GIF 39 kb)

High resolution image (EPS 941 kb)

## Abbreviations

ZIKV: Zika virus
GBS: Guillain-Barré syndrome
A129 and IFNAR^−/−^ mice: Interferon type 1 (αβ) receptor knockout mice
AG129: Interferon types 1 and 2 (αβ/γ) receptor knockout mice
DPI: Days post-infection
CMAP: Compound muscle action potential
VPS: Viral paresis scale
PBS: Phosphate buffered saline
NMJ: Neuromuscular junction
NF-H: Neurofilament-H
TMR: Tetramethylrhodamine
α-btx: α-Bungarotoxin
PBST: PBS with 0.5% Triton X-100
ROI: Region of interest
ChAT: Choline acetyltransferase
MBP: Myelin basic protein
ir: Immunoreactive
