# Supplementary figures and images for: Zika virus-induced acute myelitis and motor deficits in adult interferon αβ/γ receptor knockout mice
Source: J Neurovirol. 2018 Feb 23;24(3):273–90. doi: 10.1007/s13365-017-0595-z (PMC5992253; doi:10.1007/s13365-017-0595-z)

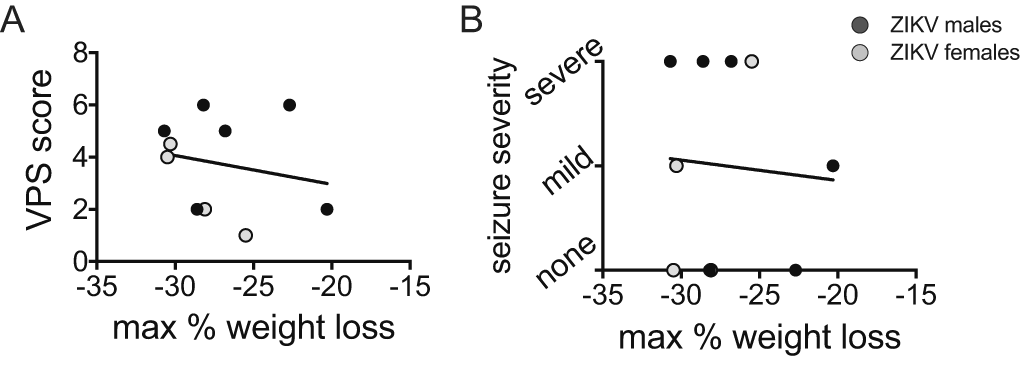

Supplement: Supplementary file 1 — Weight loss does not appear to correlate well with the severity of hindlimb deficits (A) or seizure-like activity (B). Each dot represents one ZIKV-infected animal (gray = female, black = male). Lines represent linear regression analysis. Slopes were not significantly different from zero. (GIF 18 kb) [file 13365_2017_595_Fig12_ESM.gif]

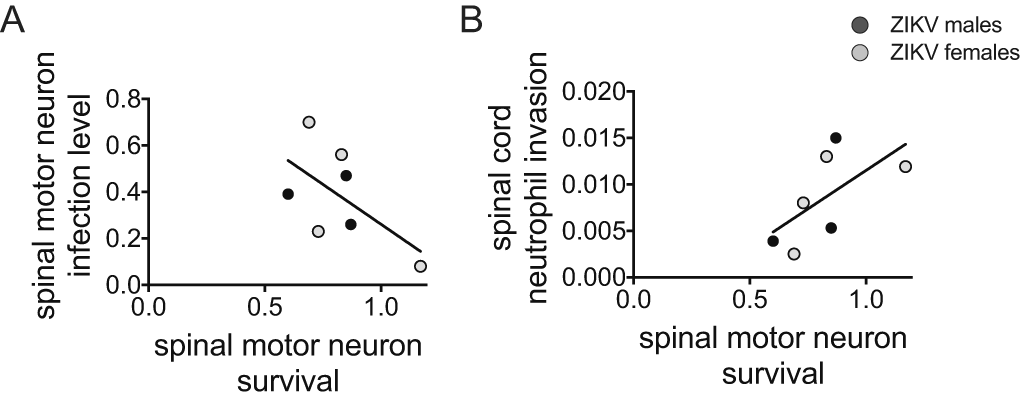

Supplement: Supplementary file 3 — Spinal motor neuron survival trends towards an association with a lower level of spinal motor neuron infection (A) and increased neutrophil invasion (B). Each dot represents one ZIKV-infected animal (gray = female, black = male). Lines represent linear regression analysis. Slopes were not significantly different from zero. (GIF 20 kb) [file 13365_2017_595_Fig13_ESM.gif]

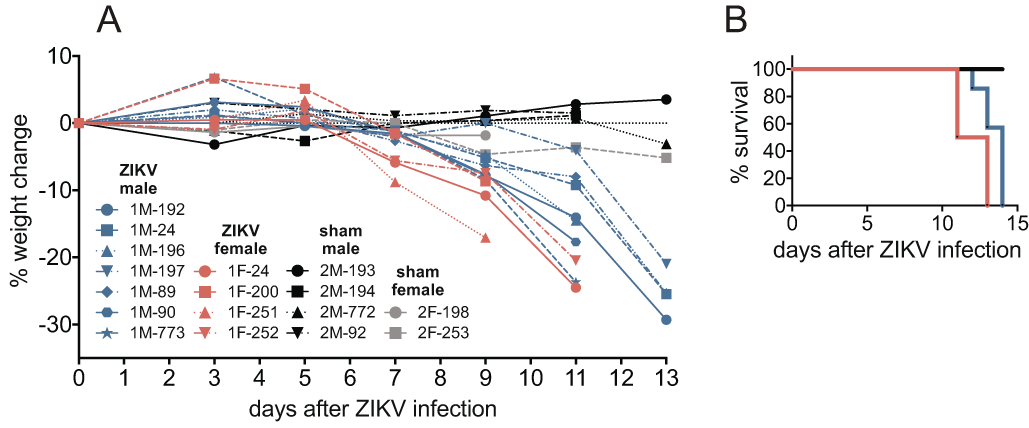

Supplement: Supplementary file 5 — Weight loss and survival curves for AG129 adult mice infected with ZIKV in Experiment #2. A, Body weight expressed in terms of percent of starting weight after ZIKV or sham infection. B, Percentage of each group surviving at each day after ZIKV or sham infection. All sham-infected animals (male and female) are represented in the sham-group. (GIF 37 kb) [file 13365_2017_595_Fig14_ESM.gif]

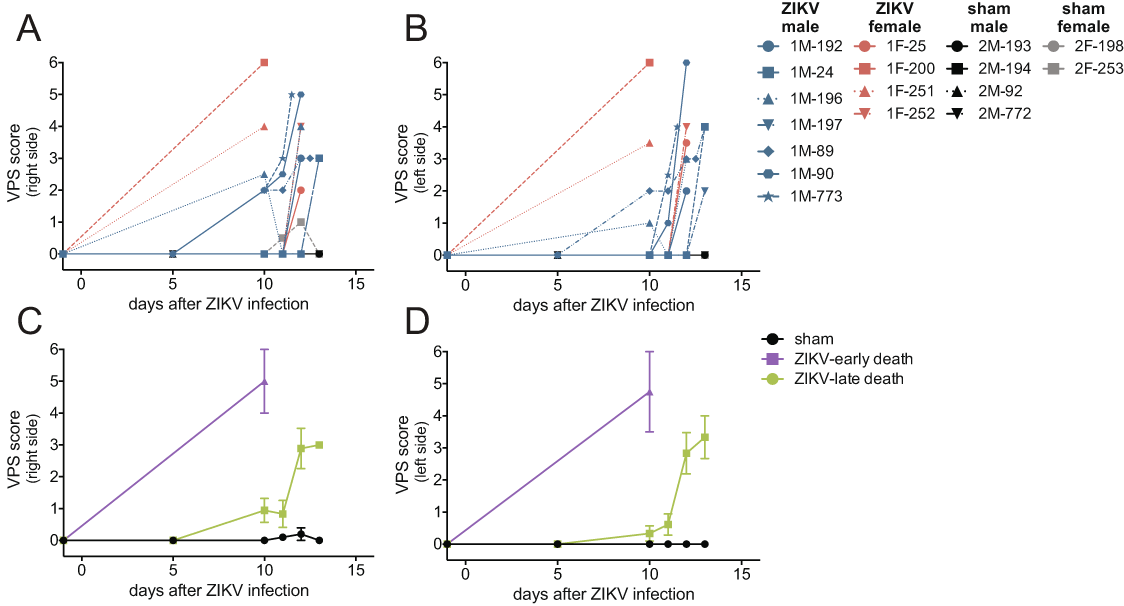

Supplement: Supplementary file 7 — Moderate paresis to severe paralysis before death in ZIKV-infected AG129 mice was reproducible. A, B, VPS scores from Experiment #2 of individual mice on the right (A) and left sides (B). C, D, Mean VPS scores of ZIKV-infected mice that died early (n = 2) or late (n = 9) compared to sham infected mice (n = 6) on the right (C) and left sides (D). Error bars represent SEM. (GIF 39 kb) [file 13365_2017_595_Fig15_ESM.gif]
